# Supplementary material for: An E280K Missense Variant in KCND3/Kv4.3—Case Report and Functional Characterization
Source: Int J Mol Sci. 2023 Jun 30;24(13):10924. doi: 10.3390/ijms241310924 (PMC10341420; doi:10.3390/ijms241310924)
Supplement: Supplementary file 1 [file ijms-24-10924-s001.zip › ijms-2420548-supplementary.pdf]

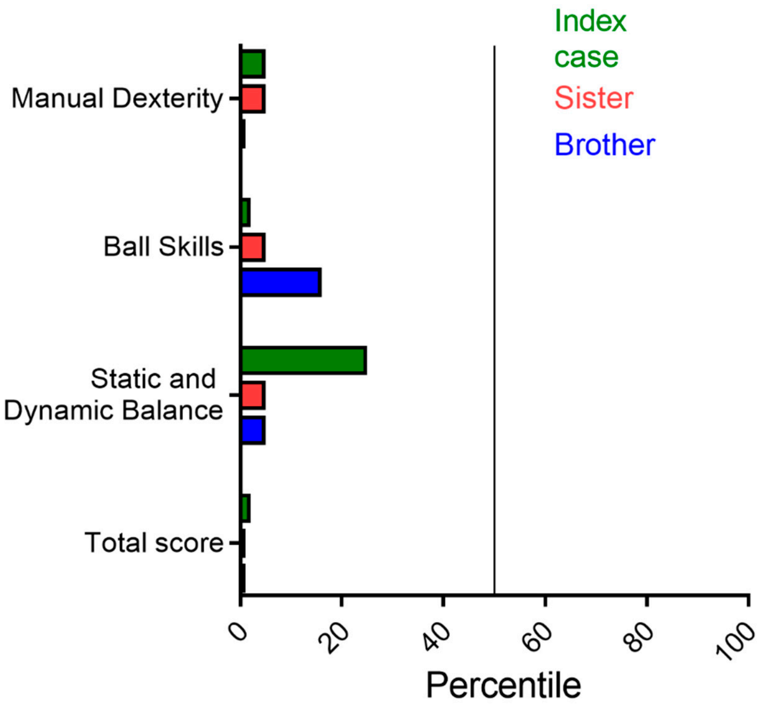

Supplementary Figure S1. Percentile scores for the three siblings in the Movement Assessment Battery for Children. Siblings were tested at five years of age.

Supplementary Table S1. Movement disorders screening panel.

Gene	Associated Phenotype description and OMIM disease ID*
AARS2	Combined oxidative phosphorylation deficiency 8, 614096<br>Leukoencephalopathy, progressive, with ovarian failure, 615889
ABCB7	Anemia, sideroblastic, with ataxia, 301310
ABCD1	Adrenomyeloneuropathy, adult, 300100<br>Adrenoleukodystrophy, 300100
ABHD12	Polyneuropathy, hearing loss, ataxia, retinitis pigmentosa, and cataract, 612674
ACTB	?Dystonia, juvenile-onset, 607371<br>Baraitser-Winter syndrome 1, 243310
ADAR	Dyschromatosis symmetrica hereditaria, 127400 Aicardi-<br>Goutieres syndrome 6, 615010
ADCY5	Dyskinesia, familial, with facial myokymia, 606703
ADGRG1	Polymicrogyria, bilateral perisylvian, 615752<br>Polymicrogyria, bilateral frontoparietal, 606854
ADPRS	Neurodegeneration, childhood-onset, stress-induced, with variable ataxia and seizures, 618170
AFG3L2	Spastic ataxia 5, autosomal recessive, 614487<br>Optic atrophy 12, 618977<br>Spinocerebellar ataxia 28, 610246
AGA	Aspartylglucosaminuria, 208400
AGTPBP1	Neurodegeneration, childhood-onset, with cerebellar atrophy, 618276
AIMP1	Leukodystrophy, hypomyelinating, 3, 260600
ALDH18A1	Cutis laxa, autosomal recessive, type IIIA, 219150<br>Cutis laxa, autosomal dominant 3, 616603<br>Spastic paraplegia 9B, autosomal recessive, 616586<br>Spastic paraplegia 9A, autosomal dominant, 601162

ALDH3A2	Sjogren-Larsson syndrome, 270200
ALDH5A1	Succinic semialdehyde dehydrogenase deficiency, 271980
ALS2	Primary lateral sclerosis, juvenile, 606353<br>Amyotrophic lateral sclerosis 2, juvenile, 205100<br>Spastic paralysis, infantile onset ascending, 607225
AMPD2	?Spastic paraplegia 63, 615686<br>Pontocerebellar hypoplasia, type 9, 615809
ANO10	Spinocerebellar ataxia, autosomal recessive 10, 613728
ANO3	Dystonia 24, 615034
AP4B1	Spastic paraplegia 47, autosomal recessive, 614066
AP4E1	Stuttering, familial persistent, 1, 184450<br>Spastic paraplegia 51, autosomal recessive, 613744
AP4M1	Spastic paraplegia 50, autosomal recessive, 612936
AP4S1	Spastic paraplegia 52, autosomal recessive, 614067
APTX	Ataxia, early-onset, with oculomotor apraxia and hypoalbuminemia, 208920
ARG1	Argininemia, 207800
ARSA	Metachromatic leukodystrophy, 250100
ARX	Lissencephaly, X-linked 2, 300215<br>Developmental and epileptic encephalopathy 1, 308350<br>Proud syndrome, 300004<br>Mental retardation, X-linked 29 and others, 300419<br>Partington syndrome, 309510<br>Hydranencephaly with abnormal genitalia, 300215
ASPA	Canavan disease, 271900
ATCAY	Ataxia, cerebellar, Cayman type, 601238
ATL1	Spastic paraplegia 3A, autosomal dominant, 182600<br>Neuropathy, hereditary sensory, type ID, 613708

ATM	Ataxia-telangiectasia, 208900<br>{Breast cancer, susceptibility to}, 114480<br>Lymphoma, mantle cell, somatic, 0<br>Lymphoma, B-cell non-Hodgkin, somatic, 0 T-<br>cell prolymphocytic leukemia, somatic, 0
ATP13A2	Kufor-Rakeb syndrome, 606693<br>Spastic paraplegia 78, autosomal recessive, 617225
ATP1A2	Migraine, familial hemiplegic, 2, 602481<br>Migraine, familial basilar, 602481<br>Alternating hemiplegia of childhood 1, 104290
ATP1A3	CAPOS syndrome, 601338<br>Alternating hemiplegia of childhood 2, 614820 Dystonia-<br>12, 128235
ATP2B3	?Spinocerebellar ataxia, X-linked 1, 302500
ATP7B	Wilson disease, 277900
B4GALNT1	Spastic paraplegia 26, autosomal recessive, 609195
BCAP31	Deafness, dystonia, and cerebral hypomyelination, 300475
BCKDHA	Maple syrup urine disease, type Ia, 248600
BCKDHB	Maple syrup urine disease, type Ib, 248600
BCL11B	Immunodeficiency 49, 617237<br>Intellectual developmental disorder with dysmorphic facies, speech delay, and T-cell abnormalities, 618092
BSCL2	Neuropathy, distal hereditary motor, type VC, 619112<br>Lipodystrophy, congenital generalized, type 2, 269700<br>Silver spastic paraplegia syndrome, 270685<br>Encephalopathy, progressive, with or without lipodystrophy, 615924
BTD	Biotinidase deficiency, 253260
C12orf65	Spastic paraplegia 55, autosomal recessive, 615035<br>Combined oxidative phosphorylation deficiency 7, 613559
C19orf12	Neurodegeneration with brain iron accumulation 4,<br>614298 ?Spastic paraplegia 43, autosomal recessive, 615043

CA8	Cerebellar ataxia and mental retardation with or without quadrupedal locomotion 3, 613227
CACNA1A	Spinocerebellar ataxia 6, 183086<br>Episodic ataxia, type 2, 108500<br>Migraine, familial hemiplegic, 1, 141500<br>Developemental and epileptic encephalopathy 42, 617106<br>Migraine, familial hemiplegic, 1, with progressive cerebellar ataxia, 141500
CACNA1E	Developmental and epileptic encephalopathy 69, 618285
CACNA1G	Spinocerebellar ataxia 42, 616795<br>Spinocerebellar ataxia 42, early-onset, severe, with neurodevelopmental deficits, 618087
CACNB4	{Epilepsy, idiopathic generalized, susceptibility to, 9}, 607682<br>{Epilepsy, juvenile myoclonic, susceptibility to, 6}, 607682<br>Episodic ataxia, type 5, 613855
CAMTA1	Cerebellar ataxia, nonprogressive, with mental retardation, 614756
CAPN1	Spastic paraplegia 76, autosomal recessive, 616907
CCT5	Neuropathy, hereditary sensory, with spastic paraplegia, 256840
CHMP1A	Pontocerebellar hypoplasia, type 8, 614961
CLCN2	{Epilepsy, juvenile absence, susceptibility to, 2}, 607628<br>{Epilepsy, idiopathic generalized, susceptibility to, 11}, 607628<br>{Epilepsy, juvenile myoclonic, susceptibility to, 8}, 607628<br>Leukoencephalopathy with ataxia, 615651<br>Hyperaldosteronism, familial, type II, 605635
CLCN4	Raynaud-Claes syndrome, 300114
CLP1	Pontocerebellar hypoplasia, type 10, 615803
CLPB	3-methylglutaconic aciduria, type VII, with cataracts, neurologic involvement and neutropenia, 616271
COASY	Neurodegeneration with brain iron accumulation 6, 615643 Pontocerebellar<br>hypoplasia, type 12, 618266

COL4A1	Angiopathy, hereditary, with nephropathy, aneurysms, and muscle cramps, 611773<br>Brain small vessel disease with or without ocular anomalies, 175780<br>{Hemorrhage, intracerebral, susceptibility to}, 614519<br>?Retinal arteries, tortuosity of, 180000<br>Microangiopathy and leukoencephalopathy, pontine, autosomal dominant, 618564
COL6A1	Ullrich congenital muscular dystrophy 1, 254090<br>Bethlem myopathy 1, 158810
COL6A2	Bethlem myopathy 1, 158810<br>Ullrich congenital muscular dystrophy 1,<br>254090 ?Myosclerosis, congenital, 255600
COL6A3	Bethlem myopathy 1, 158810<br>Dystonia 27, 616411<br>Ullrich congenital muscular dystrophy 1, 254090
COQ2	{Multiple system atrophy, susceptibility to}, 146500<br>Coenzyme Q10 deficiency, primary, 1, 607426
COQ4	Coenzyme Q10 deficiency, primary, 7, 616276
COQ8A	Coenzyme Q10 deficiency, primary, 4, 612016
COQ9	Coenzyme Q10 deficiency, primary, 5, 614654
COX20	Mitochondrial complex IV deficiency, nuclear type 11, 619054
CP	[Hypoceruloplasminemia, hereditary], 604290<br>Hemosiderosis, systemic, due to aceruloplasminemia, 604290<br>Cerebellar ataxia, 604290
CSF1R	Brain abnormalities, neurodegeneration, and dysosteosclerosis, 618476 Leukoencephalopathy,<br>diffuse hereditary, with spheroids, 221820
CSTB	Epilepsy, progressive myoclonic 1A (Unverricht and Lundborg), 254800
CTBP1	Hypotonia, ataxia, developmental delay, and tooth enamel defect syndrome, 617915
CYP27A1	Cerebrotendinous xanthomatosis, 213700
CYP2U1	Spastic paraplegia 56, autosomal recessive, 615030
CYP7B1	Spastic paraplegia 5A, autosomal recessive, 270800<br>Bile acid synthesis defect, congenital, 3, 613812

DARS1	Hypomyelination with brainstem and spinal cord involvement and leg spasticity, 615281
DARS2	Leukoencephalopathy with brain stem and spinal cord involvement and lactate elevation, 611105
DBT	Maple syrup urine disease, type II, 248600
DCAF17	Woodhouse-Sakati syndrome, 241080
DCC	Esophageal carcinoma, somatic, 133239<br>Gaze palsy, familial horizontal, with progressive scoliosis, 2, 617542<br>Mirror movements 1 and/or agenesis of the corpus callosum, 157600 Colorectal cancer, somatic, 114500
DCTN1	{Amyotrophic lateral sclerosis, susceptibility to}, 105400<br>Perry syndrome, 168605<br>Neuronopathy, distal hereditary motor, type VIIB, 607641
DDC	Aromatic L-amino acid decarboxylase deficiency, 608643
DDHD1	Spastic paraplegia 28, autosomal recessive, 609340
DDHD2	Spastic paraplegia 54, autosomal recessive, 615033
DHDDS	Retinitis pigmentosa 59, 613861<br>Developmental delay and seizures with or without movement abnormalities, 617836 ?Congenital disorder of glycosylation, type 1bb, 613861
DLAT	Pyruvate dehydrogenase E2 deficiency, 245348
DLD	Dihydrolipoamide dehydrogenase deficiency, 246900
DNAJC12	Hyperphenylalaninemia, mild, non-BH4-deficient, 617384
DNAJC3	?Ataxia, combined cerebellar and peripheral, with hearing loss and diabetes mellitus, 616192
DNAL4	?Mirror movements 3, 616059
DNM1L	Encephalopathy, lethal, due to defective mitochondrial peroxisomal fission 1, 614388<br>Optic atrophy 5, 610708
DNMT1	Cerebellar ataxia, deafness, and narcolepsy, autosomal dominant, 604121 Neuropathy, hereditary sensory, type IE, 614116
DPYS	Dihydropyrimidinuria, 222748

ECHS1	Mitochondrial short-chain enoyl-CoA hydratase 1 deficiency, 616277
EIF2B1	Leukoencephalopathy with vanishing white matter, 603896
EIF2B2	Ovarioleukodystrophy, 603896<br>Leukoencephalopathy with vanishing white matter, 603896
EIF2B3	Leukoencephalopathy with vanishing white matter, 603896
EIF2B4	Leukoencephalopathy with vanishing white matter, 603896<br>Ovarioleukodystrophy, 603896
EIF2B5	Leukoencephalopathy with vanishing white matter, 603896<br>Ovarioleukodystrophy, 603896
ELOVL4	Spinocerebellar ataxia 34, 133190<br>Stargardt disease 3, 600110<br>Ichthyosis, spastic quadriplegia, and mental retardation, 614457
ELOVL5	Spinocerebellar ataxia 38, 615957
ERLIN2	Spastic paraplegia 18, autosomal recessive, 611225
ETHE1	Ethylmalonic encephalopathy, 602473
EXOSC3	Pontocerebellar hypoplasia, type 1B, 614678
EXOSC5	No OMIM disease ID
EXOSC8	Pontocerebellar hypoplasia, type 1C, 616081
EXOSC9	Pontocerebellar hypoplasia, type 1D, 618065
FA2H	Spastic paraplegia 35, autosomal recessive, 612319
FAM126A	Leukodystrophy, hypomyelinating, 5, 610532
FAR1	Peroxisomal fatty acyl-CoA reductase 1 disorder, 616154
FARS2	Spastic paraplegia 77, autosomal recessive, 617046<br>Combined oxidative phosphorylation deficiency 14, 614946
FBXO7	Parkinson disease 15, autosomal recessive, 260300
FGF14	Spinocerebellar ataxia 27, 609307

FLVCR1	Ataxia, posterior column, with retinitis pigmentosa, 609033
FOLR1	Neurodegeneration due to cerebral folate transport deficiency, 613068
FRMD7	Nystagmus 1, congenital, X-linked, 310700<br>Nystagmus, infantile periodic alternating, X-linked, 310700
FTL	Hyperferritinemia-cataract syndrome, 600886<br>Neurodegeneration with brain iron accumulation 3, 606159 L-<br>ferritin deficiency, dominant and recessive, 615604
GALC	Krabbe disease, 245200
GAN	Giant axonal neuropathy-1, 256850
GBA	Gaucher disease, type III, 231000<br>{Parkinson disease, late-onset, susceptibility to}, 168600<br>Gaucher disease, type IIIC, 231005<br>Gaucher disease, type I, 230800<br>Gaucher disease, perinatal lethal, 608013<br>Gaucher disease, type II, 230900<br>{Lewy body dementia, susceptibility to}, 127750
GBA2	Spastic paraplegia 46, autosomal recessive, 614409
GBE1	Polyglucosan body disease, adult form, 263570<br>Glycogen storage disease IV, 232500
GCDH	Glutaricaciduria, type I, 231670
GCH1	Hyperphenylalaninemia, BH4-deficient, B, 233910<br>Dystonia, DOPA-responsive, with or without hyperphenylalaninemia, 128230
GDAP2	Spinocerebellar ataxia, autosomal recessive 27, 618369
GFAP	Alexander disease, 203450
GJC2	Spastic paraplegia 44, autosomal recessive, 613206<br>Lymphatic malformation 3, 613480<br>Leukodystrophy, hypomyelinating, 2, 608804

GLB1	GM1-gangliosidosis, type III, 230650<br>GM1-gangliosidosis, type I, 230500<br>Mucopolysaccharidosis type IVB (Morquio), 253010<br>GM1-gangliosidosis, type II, 230600
GNAL	Dystonia 25, 615073
GOSR2	Epilepsy, progressive myoclonic 6, 614018
GPR143	Nystagmus 6, congenital, X-linked, 300814<br>Ocular albinism, type I, Nettleship-Falls type, 300500
GRID2	Spinocerebellar ataxia, autosomal recessive 18, 616204
GRIN1	Neurodevelopmental disorder with or without hyperkinetic movements and seizures, autosomal recessive, 617820<br>Neurodevelopmental disorder with or without hyperkinetic movements and seizures, autosomal dominant, 614254
GRIN2B	Mental retardation, autosomal dominant 6, 613970<br>Developmental and epileptic encephalopathy 27, 616139
GRM1	Spinocerebellar ataxia 44, 617691<br>Spinocerebellar ataxia, autosomal recessive 13, 614831
HACE1	Spastic paraplegia and psychomotor retardation with or without seizures, 616756
HEXB	Sandhoff disease, infantile, juvenile, and adult forms, 268800
HK1	Hemolytic anemia due to hexokinase deficiency, 235700<br>Neuropathy, hereditary motor and sensory, Russe type, 605285<br>Neurodevelopmental disorder with visual defects and brain anomalies, 618547 Retinitis pigmentosa 79, 617460
HPDL	Spastic paraplegia 83, autosomal recessive, 619027<br>Neurodevelopmental disorder with progressive spasticity and brain white matter abnormalities, 619026
HPRT1	Hyperuricemia, HRPT-related, 300323 Lesch-Nyhan syndrome, 300322
HSD17B4	D-bifunctional protein deficiency, 261515<br>Perrault syndrome 1, 233400
HSPD1	Spastic paraplegia 13, autosomal dominant, 605280<br>Leukodystrophy, hypomyelinating, 4, 612233

IBA57	?Spastic paraplegia 74, autosomal recessive, 616451<br>Multiple mitochondrial dysfunctions syndrome 3, 615330
ISCA2	Multiple mitochondrial dysfunctions syndrome 4, 616370
ITPR1	Gillespie syndrome, 206700<br>Spinocerebellar ataxia 29, congenital nonprogressive, 117360 Spinocerebellar ataxia 15, 606658
JAM2	Basal ganglia calcification, idiopathic, 8, autosomal recessive, 618824
JAM3	Hemorrhagic destruction of the brain, subependymal calcification, and cataracts, 613730
KATNB1	Lissencephaly 6, with microcephaly, 616212
KCNA1	Episodic ataxia/myokymia syndrome, 160120
KCNA2	Developmental and epileptic encephalopathy 32, 616366
KCNC1	Epilepsy, progressive myoclonic 7, 616187
KCNC3	Spinocerebellar ataxia 13, 605259
KCND3	Brugada syndrome 9, 616399<br>Spinocerebellar ataxia 19, 607346
KCNJ10	Enlarged vestibular aqueduct, digenic, 600791<br>SESAME syndrome, 612780
KCNJ6	Keppen-Lubinsky syndrome, 614098
KCNMA1	Liang-Wang syndrome, 618729<br>{Epilepsy, idiopathic generalized, susceptibility to, 16}, 618596<br>Cerebellar atrophy, developmental delay, and seizures, 617643<br>Paroxysmal nonkinesigenic dyskinesia, 3, with or without generalized epilepsy, 609446
KCTD7	Epilepsy, progressive myoclonic 3, with or without intracellular inclusions, 611726
KIDINS220	Spastic paraplegia, intellectual disability, nystagmus, and obesity, 617296
KIF1A	NESCAV syndrome, 614255<br>Spastic paraplegia 30, autosomal dominant, 610357<br>Neuropathy, hereditary sensory, type IIC, 614213<br>Spastic paraplegia 30, autosomal recessive, 610357

KIF1C	Spastic ataxia 2, autosomal recessive, 611302
KIF5A	Myoclonus, intractable, neonatal, 617235<br>Spastic paraplegia 10, autosomal dominant, 604187<br>{Amyotrophic lateral sclerosis, susceptibility to, 25}, 617921
KMT2B	Dystonia 28, childhood-onset, 617284
L1CAM	MASA syndrome, 303350<br>Hydrocephalus with Hirschsprung disease, 307000<br>Corpus callosum, partial agenesis of, 304100<br>CRASH syndrome, 303350<br>Hydrocephalus due to aqueductal stenosis, 307000<br>Hydrocephalus with congenital idiopathic intestinal pseudoobstruction, 307000
LAMA1	Poretti-Boltshauser syndrome, 615960
LAMB1	Lissencephaly 5, 615191
LMNB1	Leukodystrophy, adult-onset, autosomal dominant, 169500
MAG	Spastic paraplegia 75, autosomal recessive, 616680
MAPK8IP3	Neurodevelopmental disorder with or without variable brain abnormalities, 618443
MARS2	Spastic ataxia 3, autosomal recessive, 611390<br>?Combined oxidative phosphorylation deficiency 25, 616430
MECP2	Encephalopathy, neonatal severe, 300673 Rett<br>syndrome, atypical, 312750<br>{Autism susceptibility, X-linked 3}, 300496<br>Rett syndrome, 312750<br>Rett syndrome, preserved speech variant, 312750<br>Mental retardation, X-linked syndromic, Lubs type, 300260<br>Mental retardation, X-linked, syndromic 13, 300055
MECR	Dystonia, childhood-onset, with optic atrophy and basal ganglia abnormalities, 617282
MFF	Encephalopathy due to defective mitochondrial and peroxisomal fission 2, 617086
MICU1	Myopathy with extrapyramidal signs, 615673
MLC1	Megalencephalic leukoencephalopathy with subcortical cysts, 604004

MMADHC	Homocystinuria, cblD type, variant 1, 277410<br>Methylmalonic aciduria and homocystinuria, cblD type, 277410<br>Methylmalonic aciduria, cblD type, variant 2, 277410
MRE11	Ataxia-telangiectasia-like disorder 1, 604391
MTHFR	{Schizophrenia, susceptibility to}, 181500<br>Homocystinuria due to MTHFR deficiency, 236250<br>{Neural tube defects, susceptibility to}, 601634<br>{Thromboembolism, susceptibility to}, 188050<br>{Vascular disease, susceptibility to}, 0
MTPAP	?Spastic ataxia 4, autosomal recessive, 613672
MTTP	Abetalipoproteinemia, 200100<br>{Metabolic syndrome, protection against}, 605552
MYORG	Basal ganglia calcification, idiopathic, 7, autosomal recessive, 618317
NANS	Spondyloepimetaphyseal dysplasia, Camera-Genevieve type, 610442
NARS2	?Deafness, autosomal recessive 94, 618434<br>Combined oxidative phosphorylation deficiency 24, 616239
NEFL	Charcot-Marie-Tooth disease, type 1F, 607734<br>Charcot-Marie-Tooth disease, dominant intermediate G, 617882 Charcot-Marie-<br>Tooth disease, type 2E, 607684
NEU1	Sialidosis, type II, 256550<br>Sialidosis, type I, 256550
NEXMIF	Mental retardation, X-linked 98, 300912
NF2	Meningioma, NF2-related, somatic, 607174<br>Schwannomatosis, somatic, 162091<br>Neurofibromatosis, type 2, 101000
NGLY1	Congenital disorder of deglycosylation, 615273
NIPA1	Spastic paraplegia 6, autosomal dominant, 600363
NKX2-1	{Thyroid cancer, nonmedullary, 1}, 188550<br>Chorea, hereditary benign, 118700<br>Choreoathetosis, hypothyroidism, and neonatal respiratory distress, 610978

NKX6-2	Spastic ataxia 8, autosomal recessive, with hypomyelinating leukodystrophy, 617560
NOL3	?Myoclonus, familial, 1, 614937
NPC1	Niemann-Pick disease, type D, 257220 Niemann-Pick disease, type C1, 257220
NPC2	Niemann-pick disease, type C2, 607625
NT5C2	Spastic paraplegia 45, autosomal recessive, 613162
NUP62	Striatonigral degeneration, infantile, 271930
OCLN	Pseudo-TORCH syndrome 1, 251290
OPA1	{Glaucoma, normal tension, susceptibility to}, 606657<br>Behr syndrome, 210000<br>Optic atrophy 1, 165500<br>Optic atrophy plus syndrome, 125250<br>?Mitochondrial DNA depletion syndrome 14 (encephalocardiomyopathic type), 616896
OPHN1	Mental retardation, X-linked, with cerebellar hypoplasia and distinctive facial appearance, 300486
PACS2	Developmental and epileptic encephalopathy 66, 618067
PANK2	HARP syndrome, 607236<br>Neurodegeneration with brain iron accumulation 1, 234200
PAX6	Optic nerve hypoplasia, 165550<br>?Coloboma, ocular, 120200<br>Foveal hypoplasia 1, 136520<br>Aniridia, 106210<br>Keratitis, 148190<br>Anterior segment dysgenesis 5, multiple subtypes, 604229<br>Cataract with late-onset corneal dystrophy, 106210<br>?Coloboma of optic nerve, 120430<br>?Morning glory disc anomaly, 120430
PCYT2	Spastic paraplegia 82, autosomal recessive, 618770
PDE10A	Dyskinesia, limb and orofacial, infantile-onset, 616921<br>Striatal degeneration, autosomal dominant, 616922

PDE8B	Striatal degeneration, autosomal dominant, 609161<br>Pigmented nodular adrenocortical disease, primary, 3, 614190
PDGFB	Dermatofibrosarcoma protuberans, 607907<br>Basal ganglia calcification, idiopathic, 5, 615483<br>Meningioma, SIS-related, 607174
PDGFRB	Myeloproliferative disorder with eosinophilia, 131440<br>Basal ganglia calcification, idiopathic, 4, 615007<br>Kosaki overgrowth syndrome, 616592<br>Premature aging syndrome, Penttinen type, 601812<br>Myofibromatosis, infantile, 1, 228550
PDHA1	Pyruvate dehydrogenase E1-alpha deficiency, 312170
PDHX	Lacticacidemia due to PDX1 deficiency, 245349
PDSS1	Coenzyme Q10 deficiency, primary, 2, 614651
PDSS2	Coenzyme Q10 deficiency, primary, 3, 614652
PDYN	Spinocerebellar ataxia 23, 610245
PEX10	Peroxisome biogenesis disorder 6B, 614871<br>Peroxisome biogenesis disorder 6A (Zellweger), 614870
PEX2	Peroxisome biogenesis disorder 5A (Zellweger), 614866<br>Peroxisome biogenesis disorder 5B, 614867
PEX7	Peroxisome biogenesis disorder 9B, 614879<br>Rhizomelic chondrodysplasia punctata, type 1, 215100
PHYH	Refsum disease, 266500
PIK3R5	Ataxia-oculomotor apraxia 3, 615217
PLA2G6	Infantile neuroaxonal dystrophy 1, 256600<br>Parkinson disease 14, autosomal recessive, 612953<br>Neurodegeneration with brain iron accumulation 2B, 610217
PLP1	Pelizaeus-Merzbacher disease, 312080<br>Spastic paraplegia 2, X-linked, 312920
PMM2	Congenital disorder of glycosylation, type Ia, 212065

PMP22	Dejerine-Sottas disease, 145900<br>?Neuropathy, inflammatory demyelinating, 139393<br>Charcot-Marie-Tooth disease, type 1E, 118300<br>Roussy-Levy syndrome, 180800<br>Neuropathy, recurrent, with pressure palsies, 162500<br>Charcot-Marie-Tooth disease, type 1A, 118220
PMPCA	Spinocerebellar ataxia, autosomal recessive 2, 213200
PNKD	Paroxysmal nonkinesigenic dyskinesia 1, 118800
PNKP	Microcephaly, seizures, and developmental delay, 613402<br>Ataxia-oculomotor apraxia 4, 616267<br>?Charcot-Marie-Tooth disease, type 2B2, 605589
PNPLA6	Spastic paraplegia 39, autosomal recessive, 612020<br>Boucher-Neuhauser syndrome, 215470<br>Oliver-McFarlane syndrome, 275400<br>?Laurence-Moon syndrome, 245800
POLG	Progressive external ophthalmoplegia, autosomal dominant 1, 157640<br>Mitochondrial DNA depletion syndrome 4B (MNGIE type), 613662<br>Mitochondrial recessive ataxia syndrome (includes SANDO and SCAE), 607459<br>Mitochondrial DNA depletion syndrome 4A (Alpers type), 203700<br>Progressive external ophthalmoplegia, autosomal recessive 1, 258450
POLR1C	Treacher Collins syndrome 3, 248390<br>Leukodystrophy, hypomyelinating, 11, 616494
POLR3A	Leukodystrophy, hypomyelinating, 7, with or without oligodontia and/or hypogonadotropic hypogonadism, 607694<br>Wiedemann-Rautenstrauch syndrome, 264090
POLR3B	Leukodystrophy, hypomyelinating, 8, with or without oligodontia and/or hypogonadotropic hypogonadism, 614381
PRF1	Hemophagocytic lymphohistiocytosis, familial, 2, 603553<br>Aplastic anemia, 609135<br>Lymphoma, non-Hodgkin, 605027
PRICKLE1	Epilepsy, progressive myoclonic 1B, 612437
PRKCG	Spinocerebellar ataxia 14, 605361

PRKRA	Dystonia 16, 612067
PRRT2	Episodic kinesigenic dyskinesia 1, 128200<br>Seizures, benign familial infantile, 2, 605751<br>Convulsions, familial infantile, with paroxysmal choreoathetosis, 602066
PSAP	Gaucher disease, atypical, 610539<br>Krabbe disease, atypical, 611722<br>Combined SAP deficiency, 611721<br>Metachromatic leukodystrophy due to SAP-b deficiency, 249900
PTRH2	Infantile-onset multisystem neurologic, endocrine, and pancreatic disease, 616263
PTS	Hyperphenylalaninemia, BH4-deficient, A, 261640
PUM1	Spinocerebellar ataxia 47, 617931
PYCR2	Leukodystrophy, hypomyelinating, 10, 616420
QDPR	Hyperphenylalaninemia, BH4-deficient, C, 261630
RAB18	Warburg micro syndrome 3, 614222
RAB3GAP1	Warburg micro syndrome 1, 600118
RAB3GAP2	Warburg micro syndrome 2, 614225<br>Martsolf syndrome, 212720
RAD51	{Breast cancer, susceptibility to}, 114480<br>Mirror movements 2, 614508<br>Fanconi anemia, complementation group R, 617244
RARS1	Leukodystrophy, hypomyelinating, 9, 616140
RARS2	Pontocerebellar hypoplasia, type 6, 611523
REEP1	Spastic paraplegia 31, autosomal dominant, 610250<br>?Neuronopathy, distal hereditary motor, type VB, 614751
RNASEH2A	Aicardi-Goutieres syndrome 4, 610333
RNASEH2B	Aicardi-Goutieres syndrome 2, 610181
RNASEH2C	Aicardi-Goutieres syndrome 3, 610329

RNF170	Ataxia, sensory, 1, autosomal dominant, 608984
RNF216	Cerebellar ataxia and hypogonadotropic hypogonadism, 212840
RTN2	Spastic paraplegia 12, autosomal dominant, 604805
RUBCN	Spinocerebellar ataxia, autosomal recessive 15, 615705
SACS	Spastic ataxia, Charlevoix-Saguenay type, 270550
SAMD9L	Monosomy 7 myelodysplasia and leukemia syndrome 1, 252270 Ataxia-pancytopenia syndrome, 159550
SAMHD1	?Chilblain lupus 2, 614415<br>Aicardi-Goutieres syndrome 5, 612952
SCN11A	Neuropathy, hereditary sensory and autonomic, type VII, 615548<br>Episodic pain syndrome, familial, 3, 615552
SCN1A	Febrile seizures, familial, 3A, 604403<br>Migraine, familial hemiplegic, 3, 609634<br>Dravet syndrome, 607208<br>Epilepsy, generalized, with febrile seizures plus, type 2, 604403
SCN2A	Episodic ataxia, type 9, 618924<br>Developmental and epileptic encephalopathy 11, 613721<br>Seizures, benign familial infantile, 3, 607745
SCN8A	Seizures, benign familial infantile, 5, 617080<br>Developmental and epileptic encephalopathy 13, 614558<br>Cognitive impairment with or without cerebellar ataxia, 614306<br>?Myoclonus, familial, 2, 618364
SEPSECS	Pontocerebellar hypoplasia type 2D, 613811
SERAC1	3-methylglutaconic aciduria with deafness, encephalopathy, and Leigh-like syndrome, 614739
SETX	Spinocerebellar ataxia, autosomal recessive, with axonal neuropathy 2, 606002<br>Amyotrophic lateral sclerosis 4, juvenile, 602433
SGCE	Dystonia-11, myoclonic, 159900
SIL1	Marinesco-Sjogren syndrome, 248800
SLC12A6	Agenesis of the corpus callosum with peripheral neuropathy, 218000

SLC16A2	Allan-Herndon-Dudley syndrome, 300523
SLC19A3	Thiamine metabolism dysfunction syndrome 2 (biotin- or thiamine-responsive encephalopathy type 2), 607483
SLC1A3	Episodic ataxia, type 6, 612656
SLC20A2	Basal ganglia calcification, idiopathic, 1, 213600
SLC25A15	Hyperornithinemia-hyperammonemia-homocitrullinemia syndrome, 238970
SLC2A1	Dystonia 9, 601042<br>GLUT1 deficiency syndrome 1, infantile onset, severe, 606777<br>Stomatin-deficient cryohydrocytosis with neurologic defects, 608885<br>GLUT1 deficiency syndrome 2, childhood onset, 612126<br>{Epilepsy, idiopathic generalized, susceptibility to, 12}, 614847
SLC30A10	Hypermanganesemia with dystonia 1, 613280
SLC33A1	Spastic paraplegia 42, autosomal dominant, 612539<br>Congenital cataracts, hearing loss, and neurodegeneration, 614482
SLC39A14	?Hyperostosis cranalis interna, 144755<br>Hypermanganesemia with dystonia 2, 617013
SLC52A2	Brown-Vialetto-Van Laere syndrome 2, 614707
SLC52A3	Brown-Vialetto-Van Laere syndrome 1, 211530 ?Fazio-<br>Londe disease, 211500
SLC6A3	{Nicotine dependence, protection against}, 188890<br>Parkinsonism-dystonia, infantile, 1, 613135
SLC9A1	Lichtenstein-Knorr syndrome, 616291
SMDT1	No OMIM disease ID
SMPD1	Niemann-Pick disease, type A, 257200 Niemann-<br>Pick disease, type B, 607616
SNCA	Dementia, Lewy body, 127750<br>Parkinson disease 1, 168601<br>Parkinson disease 4, 605543
SNORD118	Leukoencephalopathy, brain calcifications, and cysts, 614561

SNX14	Spinocerebellar ataxia, autosomal recessive 20, 616354
SOX10	Waardenburg syndrome, type 2E, with or without neurologic involvement, 611584<br>PCWH syndrome, 609136<br>Waardenburg syndrome, type 4C, 613266
SPART	Troyer syndrome, 275900
SPAST	Spastic paraplegia 4, autosomal dominant, 182601
SPG11	Charcot-Marie-Tooth disease, axonal, type 2X, 616668<br>Spastic paraplegia 11, autosomal recessive, 604360<br>Amyotrophic lateral sclerosis 5, juvenile, 602099
SPG21	Mast syndrome, 248900
SPG7	Spastic paraplegia 7, autosomal recessive, 607259
SPR	Dystonia, dopa-responsive, due to sepiapterin reductase deficiency, 612716
SPTBN2	Spinocerebellar ataxia, autosomal recessive 14, 615386<br>Spinocerebellar ataxia 5, 600224
STUB1	Spinocerebellar ataxia, autosomal recessive 16, 615768 ?Spinocerebellar ataxia 48, 618093
SUMF1	Multiple sulfatase deficiency, 272200
SUOX	Sulfite oxidase deficiency, 272300
SYNE1	Arthrogryposis multiplex congenita 3, myogenic type, 618484<br>Spinocerebellar ataxia, autosomal recessive 8, 610743<br>Emery-Dreifuss muscular dystrophy 4, autosomal dominant, 612998
TAF1	Dystonia-Parkinsonism, X-linked, 314250<br>Mental retardation, X-linked, syndromic 33, 300966
TANGO2	Metabolic encephalomyopathic crises, recurrent, with rhabdomyolysis, cardiac arrhythmias, and neurodegeneration, 616878
TBC1D20	Warburg micro syndrome 4, 615663
TBC1D23	Pontocerebellar hypoplasia, type 11, 617695
TBCD	Encephalopathy, progressive, early-onset, with brain atrophy and thin corpus callosum, 617193

TDP1	?Spinocerebellar ataxia, autosomal recessive, with axonal neuropathy 1, 607250
TDP2	Spinocerebellar ataxia, autosomal recessive 23, 616949
TECPR2	Spastic paraplegia 49, autosomal recessive, 615031
TENM4	Essential tremor, hereditary, 5, 616736
TGM6	Spinocerebellar ataxia 35, 613908
TH	Segawa syndrome, recessive, 605407
THAP1	Dystonia 6, torsion, 602629
TIMM8A	Mohr-Tranebjaerg syndrome, 304700
TMEM106B	Leukodystrophy, hypomyelinating, 16, 617964
TMEM240	Spinocerebellar ataxia 21, 607454
TMEM67	Meckel syndrome 3, 607361<br>COACH syndrome 1, 216360<br>?RHYNS syndrome, 602152<br>Nephronophthisis 11, 613550<br>{Bardet-Biedl syndrome 14, modifier of}, 615991<br>Joubert syndrome 6, 610688
TOE1	Pontocerebellar hypoplasia, type 7, 614969
TOR1A	Dystonia-1, torsion, 128100<br>Arthrogryposis multiplex congenita 5, 618947<br>{Dystonia-1, modifier of}, 0
TPP1	Spinocerebellar ataxia, autosomal recessive 7, 609270<br>Ceroid lipofuscinosis, neuronal, 2, 204500
TREM2	Polycystic lipomembranous osteodysplasia with sclerosing leukoencephalopathy 2, 618193
TREX1	{Systemic lupus erythematosus, susceptibility to}, 152700<br>Vasculopathy, retinal, with cerebral leukodystrophy, 192315<br>Aicardi-Goutieres syndrome 1, dominant and recessive, 225750<br>Chilblain lupus, 610448

TRPM3	No OMIM disease ID
TSEN15	Pontocerebellar hypoplasia, type 2F, 617026
TSEN2	Pontocerebellar hypoplasia type 2B, 612389
TSEN54	Pontocerebellar hypoplasia type 4, 225753<br>Pontocerebellar hypoplasia type 2A, 277470<br>?Pontocerebellar hypoplasia type 5, 610204
TTBK2	Spinocerebellar ataxia 11, 604432
TTC19	Mitochondrial complex III deficiency, nuclear type 2, 615157
TTPA	Ataxia with isolated vitamin E deficiency, 277460
TUBA1A	Lissencephaly 3, 611603
TUBB	Symmetric circumferential skin creases, congenital, 1, 156610<br>Cortical dysplasia, complex, with other brain malformations 6, 615771
TUBB4A	Leukodystrophy, hypomyelinating, 6, 612438<br>Dystonia 4, torsion, autosomal dominant, 128101
TUBG1	Cortical dysplasia, complex, with other brain malformations 4, 615412
TWNK	Mitochondrial DNA depletion syndrome 7 (hepatocerebral type), 271245<br>Progressive external ophthalmoplegia with mitochondrial DNA deletions, autosomal dominant 3, 609286<br>Perrault syndrome 5, 616138
TYROBP	Polycystic lipomembranous osteodysplasia with sclerosing leukoencephalopathy 1, 221770
UBAP1	Spastic paraplegia 80, autosomal dominant, 618418
UBTF	Neurodegeneration, childhood-onset, with brain atrophy, 617672
VAMP1	Spastic ataxia 1, autosomal dominant, 108600<br>Myasthenic syndrome, congenital, 25, 618323
VARS2	Combined oxidative phosphorylation deficiency 20, 615917
VCP	Inclusion body myopathy with early-onset Paget disease and frontotemporal dementia 1, 167320 Charcot-Marie-Tooth disease, type 2Y, 616687<br>Frontotemporal dementia and/or amyotrophic lateral sclerosis 6, 613954

VLDLR	Cerebellar hypoplasia and mental retardation with or without quadrupedal locomotion 1, 224050
VPS11	Leukodystrophy, hypomyelinating, 12, 616683
VPS13A	Choreoacanthocytosis, 200150
VPS13D	Spinocerebellar ataxia, autosomal recessive 4, 607317
VPS16	No OMIM disease ID
VPS37A	Spastic paraplegia 53, autosomal recessive, 614898
VPS53	Pontocerebellar hypoplasia, type 2E, 615851
VRK1	Pontocerebellar hypoplasia type 1A, 607596
WASHC5	Ritscher-Schinzel syndrome 1, 220210<br>Spastic paraplegia 8, autosomal dominant, 603563
WDR26	Skraban-Deardorff syndrome, 617616
WDR45	Neurodegeneration with brain iron accumulation 5, 300894
WDR73	Galloway-Mowat syndrome 1, 251300
WDR81	Hydrocephalus, congenital, 3, with brain anomalies, 617967<br>Cerebellar ataxia, mental retardation, and dysequilibrium syndrome 2, 610185
WWOX	Spinocerebellar ataxia, autosomal recessive 12, 614322<br>Esophageal squamous cell carcinoma, somatic, 133239<br>Developmental and epileptic encephalopathy 28, 616211
XK	McLeod syndrome with or without chronic granulomatous disease, 300842
XPR1	Basal ganglia calcification, idiopathic, 6, 616413
XRCC1	?Spinocerebellar ataxia, autosomal recessive 26, 617633
ZC4H2	Wieacker-Wolff syndrome, 314580<br>Wieacker-Wolff syndrome, female-restricted, 301041
ZFYVE26	Spastic paraplegia 15, autosomal recessive, 270700
ZFYVE27	Spastic paraplegia 33, autosomal dominant, 610244

ZNF592	No OMIM disease ID

*Disease IDs and phenotype descriptions according to OMIM, release version November 20th, 2020.
"No OMIM Disease ID" indicates that the gene lacks a disease description in OMIM. "{…}" indicates that the gene has variants that are risk factors for the described condition.
